# Efficacy and safety of artemether–lumefantrine, artesunate–amodiaquine, and dihydroartemisinin–piperaquine for the treatment of uncomplicated *Plasmodium falciparum* malaria in three provinces in Angola, 2017

**DOI:** 10.1186/s12936-018-2290-9

**Published:** 2018-04-03

**Authors:** Elizabeth Davlantes, Pedro Rafael Dimbu, Carolina Miguel Ferreira, Maria Florinda Joao, Dilunvuidi Pode, Jacinto Félix, Edgar Sanhangala, Benjamin Nieto Andrade, Samaly dos Santos Souza, Eldin Talundzic, Venkatachalam Udhayakumar, Chantelle Owens, Eliane Mbounga, Lubbe Wiesner, Eric S. Halsey, José Franco Martins, Filomeno Fortes, Mateusz M. Plucinski

**Affiliations:** 1 Epidemic Intelligence Service, United States Centers for Disease Control and Prevention, 1600 Clifton Road, Atlanta, GA 30333 USA; 2 National Malaria Control Programme, Ministry of Health, Luanda, Angola; 3 PSI Angola, Luanda, Angola; 4 Field Epidemiology Training Programme, Ministry of Health, Luanda, Angola; 5 Malaria Branch, Centers for Disease Control and Prevention, Atlanta, GA USA; 6 United States President’s Malaria Initiative, Centers for Disease Control and Prevention, Atlanta, GA USA; 7 United States President’s Malaria Initiative, United States Agency for International Development, Luanda, Angola; 8 Division of Clinical Pharmacology, University of Cape Town, Cape Town, South Africa

**Keywords:** Antimalarial resistance, Artemether–lumefantrine, Artesunate–amodiaquine, Dihydroartemisinin–piperaquine, *pfK13*, *pfmdr1*, *pfcrt*, *pfpm2*

## Abstract

**Background:**

The Angolan government recommends three artemisinin-based combinations for the treatment of uncomplicated *Plasmodium falciparum* malaria: artemether–lumefantrine (AL), artesunate–amodiaquine (ASAQ), and dihydroartemisinin–piperaquine (DP). Due to the threat of emerging anti-malarial drug resistance, it is important to periodically monitor the efficacy of artemisinin-based combination therapy (ACT). This study evaluated these medications’ therapeutic efficacy in Benguela, Lunda Sul, and Zaire Provinces.

**Methods:**

Enrollment occurred between March and July 2017. Study participants were children with *P. falciparum* monoinfection from each provincial capital. Participants received a 3-day course of a quality-assured artemisinin-based combination and were monitored for 28 (AL and ASAQ arms) or 42 days (DP arm). Each ACT was assessed in two provinces. The primary study endpoints were: (1) follow-up without complications and (2) failure to respond to treatment or development of recurrent *P. falciparum* infection. Parasites from each patient experiencing recurrent infection were genotyped to differentiate new infection from recrudescence of persistent parasitaemia. These parasites were also analysed for molecular markers associated with ACT resistance.

**Results:**

Of 608 children enrolled in the study, 540 (89%) reached a primary study endpoint. Parasitaemia was cleared within 3 days of medication administration in all participants, and no early treatment failures were observed. After exclusion of reinfections, the corrected efficacy of AL was 96% (91–100%, 95% confidence interval) in Zaire and 97% (93–100%) in Lunda Sul. The corrected efficacy of ASAQ was 100% (97–100%) in Benguela and 93% (88–99%) in Zaire. The corrected efficacy of DP was 100% (96–100%) in Benguela and 100% in Lunda Sul. No mutations associated with artemisinin resistance were identified in the *pfk13* gene in the 38 cases of recurrent *P. falciparum* infection. All 33 treatment failures in the AL and ASAQ arms carried *pfmdr1* or *pfcrt* mutations associated with lumefantrine and amodiaquine resistance, respectively, on day of failure.

**Conclusions:**

AL, ASAQ, and DP continue to be efficacious against *P. falciparum* malaria in these provinces of Angola. Rapid parasite clearance and the absence of genetic evidence of artemisinin resistance are consistent with full susceptibility to artemisinin derivatives. Periodic monitoring of in vivo drug efficacy remains a priority routine activity for Angola.

**Electronic supplementary material:**

The online version of this article (10.1186/s12936-018-2290-9) contains supplementary material, which is available to authorized users.

## Background

Malaria is the principal cause of morbidity and mortality in Angola and is endemic throughout the country. The disease led to more than three million cases and 7999 deaths nationwide in 2015. In recent years, the country has made significant gains in reducing malaria burden with aggressive preventive measures, case management, and surveillance [1].

There are currently three medications equally recommended by the Angolan Ministry of Health for treatment of uncomplicated malaria: artemether–lumefantrine (AL), artesunate–amodiaquine (ASAQ), and dihydroartemisinin–piperaquine (DP). These are all artemisinin-based combinations, the drug class recommended as first-line malaria treatment by the World Health Organization (WHO) [2]. Artemisinin-based combination therapy (ACT) comprises an artemisinin derivative plus a partner drug; the artemisinin component has a relatively short half-life and acts quickly to reduce parasite burden, while the partner drugs have longer half-lives and suppress parasitaemia for weeks post-treatment. These pharmacological differences are important when monitoring for drug resistance—inadequate clearance of parasitaemia in the first few days after treatment signals a possible problem with the artemisinin component of an ACT, while recurrent parasitaemia later on points more towards an issue with the partner drug.

WHO recommends biennial surveillance of ACT efficacy to provide an early warning against the emergence and spread of resistance [3]. In Angola, the findings of such surveillance have generated international interest and demonstrate the need for continued monitoring [4]. A study of anti-malarial therapeutic efficacy in Angola in 2002–2003 revealed an efficacy of less than 20% for chloroquine [5] and encouraged the Ministry of Health to transition to ACT for first-line treatment of uncomplicated malaria [1]. Efficacy studies in 2013 and 2015 raised concerns about possible lumefantrine resistance, particularly in Zaire Province where corrected efficacy was below the 90% WHO threshold for changing treatment policy [6, 7]. Another therapeutic efficacy study using data from 2011 to 2013 prompted similar concern in Luanda [8].

Although artemisinin resistance has not yet been documented in Africa, it is widespread in Southeast Asia [9, 10]. In 2013, a Vietnamese man with recent travel to Angola’s Lunda Sul Province was diagnosed with malaria which failed to respond to repeated dosing of artemisinin derivatives [11]. Ultimately, this case was inconclusive for artemisinin failure, largely because of uncertainty around the quality of medications administered and the role of the patient’s functional asplenia in delayed parasite clearance [12, 13]. However, the case does emphasize the importance of continued surveillance, particularly as Africa and Asia forge closer economic links. The extensive use of ACT worldwide also provides a strong selective pressure for artemisinin resistance.

Another important component of anti-malarial resistance monitoring is surveillance for molecular markers of resistance among *Plasmodium falciparum* parasites. Numerous polymorphisms in the *P. falciparum* genome have been suggested to provide resistance to ACT, both to the artemisinin component and to various partner drugs. Certain mutations in the propeller domain of *pfk13*, for instance, have been shown to confer artemisinin resistance [14]. Polymorphisms in the chloroquine resistance transporter gene *pfcrt* [15], originally identified as a marker of chloroquine resistance, have also been associated with resistance to multiple drugs, including amodiaquine and lumefantrine. Polymorphisms in the gene encoding the multidrug resistance 1 transporter (*pfmdr1*) have been preliminarily associated with both lumefantrine and amodiaquine resistance, with certain mutations such as N86Y having an opposite effect on lumefantrine and amodiaquine susceptibility [16, 17]. Elevated *pfmdr1* copy number has been postulated to confer resistance to lumefantrine [18], and, most recently, elevated copy number of the plasmepsin 2 gene (*pfpm2*) has been proposed as a marker for piperaquine resistance [19, 20].

This report presents the results of the latest round of therapeutic efficacy monitoring of anti-malarials in Angola’s three fixed anti-malarial surveillance sentinel sites.

## Methods

### Study design

An in vivo assessment of the therapeutic efficacy of AL, ASAQ, and DP was conducted according to the standard WHO protocol [3]. Study participants were recently febrile children with *P. falciparum* monoinfection. Participants were followed with physical exams and blood samples for 28–42 days after anti-malarial administration for development of adverse medication effects or recurrent parasitaemia. Molecular analyses were performed on samples from those experiencing treatment failure.

### Study population

**Fig. 1:**
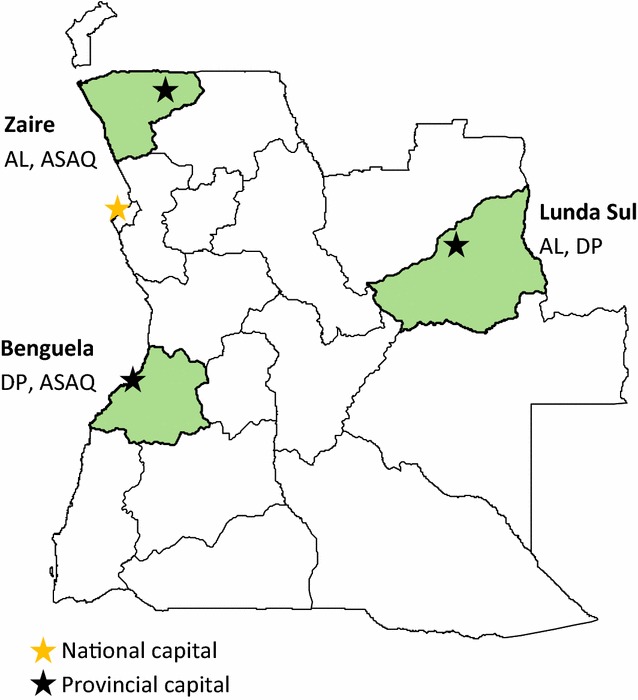
Location of sentinel sites for therapeutic efficacy monitoring in Angola, 2017

The study took place at anti-malarial resistance sentinel sites in Benguela, Lunda Sul, and Zaire that were retained from previous therapeutic efficacy studies [6, 7]. Benguela is a southern coastal province with stable mesoendemic malaria transmission. Zaire is a forested province on the northern coast that also has stable mesoendemic transmission. Lunda Sul province, part of Angola’s eastern savannah, features hyperendemic transmission (Fig. 1) [1].

Enrollment was divided into six arms, and efficacy of each medication was assessed in two provinces. Target enrollment of one hundred participants per arm provided enough power to estimate efficacy with 95% confidence limits of ± 5%, assuming an expected efficacy of 95% and a maximum loss to follow-up and exclusion rate of 27%. AL and DP were evaluated in Lunda Sul, AL and ASAQ in Zaire, and DP and ASAQ in Benguela (Fig. 1). Two drugs were assessed sequentially in each site. Medication administration was not randomized or blinded; the first hundred participants enrolled at each site received the first ACT to be evaluated there (AL in Zaire and Lunda Sul or DP in Benguela), and the second hundred enrollees received the second drug under evaluation (ASAQ in Benguela and Zaire or DP in Lunda Sul).

Screening for participants took place at two health facilities in each province’s capital from March to July 2017. Study entrants were children with fever (≥ 37.5 °C) or a history of fever in the past 24 h, *P. falciparum* monoinfection on blood smear, no signs of severe malaria on physical exam, no concomitant illness or severe malnutrition, a haemoglobin greater than 8 g/dL, no anti-malarials within the last 14 days, and caregivers willing to attend all follow-up visits with study staff. Inclusion criteria were broader in Benguela compared to Lunda Sul and Zaire, allowing for older children (< 12 years vs. < 5 years) and lower parasitaemias (between 1000 and 100,000 asexual parasites/μL vs. between 2000 and 200,000 parasites/μL), due to the lower level of malaria transmission in Benguela.

### Clinical monitoring

Enrolled children received a 3-day course of one of the anti-malarials under evaluation, dosed according to guidelines from the drug manufacturers. Quality-controlled AL (Ipca Laboratories, Maharashtra, India), ASAQ (Sanofi Aventis, Paris, France), and DP (Eurartesim^®^; Leadiant Biosciences, Rome, Italy) were provided by WHO. For ASAQ and DP, which have once-daily dosing, all three doses were given under direct observation of study staff. For AL, which requires twice-daily dosing, each morning dose was directly observed. Evening doses were given at home, and as in previous studies, efforts were made to ensure correct administration of each dose. Parents or guardians were given the appropriate weight-based dose, called at home to confirm delivery of the dose, and instructed to bring the empty pill packages back to study staff the following morning. All AL doses were administered with a snack, containing at least 3 g of fat, to facilitate drug absorption. Parents or guardians were given individual packages of yogurt or chocolate milk to take home and were asked to bring the empty package back to study staff the following morning.

After each medication administration, children were observed for 1 h to monitor for vomiting or other side effects, including diarrhea, nausea, or excessive sweating. Children who vomited within half an hour after medication administration were given a repeat dose. Children who vomited between half an hour and 1 h after medication administration were given a repeat dose that was half of the original dose. Those with persistent vomiting were excluded from the study.

Participants made eight or ten visits to study staff over the course of 28 or 42 days. Participants in the AL and ASAQ arms were followed for 28 days, while those in the DP arms required 42-day follow-up due to the longer half-life of piperaquine [3]. All children were monitored daily during the 3 days of drug administration and 1 day following; afterwards, follow-up visits occurred weekly. An interval history, physical exam, thick and thin blood smear, and dried blood spot collection were performed at each visit, except for day 1 of follow-up, in which blood samples were only collected if a child exhibited signs of severe malaria. Participants’ haemoglobin was also measured every 2 weeks. Finally, participants in the AL arm in Zaire underwent an additional dried blood spot collection on day 7 of follow-up to measure blood lumefantrine levels, to provide further insight into the large number of late treatment failures previously observed with AL in this province [6, 7].

Children were excluded from follow-up if they developed a non-falciparum malaria infection, showed signs of severe illness, missed a follow-up visit, or took another anti-malarial. For the preliminary analysis, the primary outcomes were adequate clinical and parasitological response, failure to adequately clear parasitaemia, or recurrent *P. falciparum* parasitaemia. Participants were categorized as early treatment failures if their parasitaemia increased from day 0 to day 2 of follow-up, failed to decrease by at least 75% by day 3, was associated with fever on day 3, or was associated with signs of severe malaria on days 1–3. Participants who developed any parasitaemia after day 3 were classified as late treatment failures.

### Molecular analysis

Microsatellite analysis and Sanger sequencing of *P. falciparum* parasites were performed on selected blood samples from study participants. Day 0 and day of failure samples from all cases of late treatment failure were genotyped by microsatellite analysis for reclassification as reinfection or recrudescence. Additional day 0 samples from participants not experiencing treatment failure were randomly selected for neutral microsatellite analysis to augment the number of samples for determination of allele frequencies. day 0 and day of failure samples from all cases of late treatment failure were also assessed for *pfK13, pfcrt*, and *pfmdr1* sequence and *pfmdr1* and *pfpm2* copy number.

Genomic DNA was extracted using the QIAamp blood minikit (Qiagen Inc., California, USA). Seven neutral microsatellite loci spanning six chromosomes (TA1, chromosome 6; poly α, chromosome 4; PfPK2, chromosome 12; TA109, chromosome 6; and 2490, chromosome 10; C2M24, chromosome 2; and C3M69, chromosome 3) were amplified by non-nested or semi-nested PCR and their fragment lengths were measured [21–24]. Fragments of the *pfcrt* [25], *pfmdr1* [25], and *pfK13* [26] genes were amplified using previously published primers. Direct Sanger sequencing of the nested purified PCR products was performed by using a BigDye Terminator v3.1 cycle sequencing kit on an iCycler thermal cycler (Bio-Rad, California, USA). Sequence analysis was performed by using Geneious R7 (Biomatters, Auckland, New Zealand).

*Pfmdr1* copy number was assessed using a previously described protocol [27]. The *P. falciparum β*-*tubulin* gene was used as the reference gene for relative quantification of *pfmdr1* gene copy number. Each run included the 3D7 parasite strain as a single-copy control, the W2Mef strain (two copies), and the Dd2 strain (three to four copies). The assay was performed using the Agilent Mx3005 real-time PCR instrument (Agilent Technologies, California, USA). The copy number was determined by using the relative quantification module in MxPro3005 software (Agilent Technologies, California, USA), using the comparative ΔΔCT method, and rounded to the nearest integer.

For *pfpm2* copy number analysis, the *P. falciparum β*-*tubulin* gene was again used for reference. The reverse primers of both the *pfpm2* and *β*-*tubulin* genes were modified with the PET tag and labeled with FAM (*pfpm2*) and HEX (*β*-*tubulin*) fluorophores. The PET-PCR assays were performed using Agilent Mx3005pro thermocyclers (Agilent Technologies, California, USA).

### Lumefantrine level measurement

Additional dried blood spots were collected on day 7 of follow-up from participants in the AL arm in Zaire. A volume of 50 μL of whole blood was collected on filter paper pre-treated with 0.75 M tartaric acid and stored at room temperature. Dried blood spots were eluted with an acidified acetonitrile solution followed by solid phase extraction. The extraction procedure was followed by liquid chromatographic separation using a Synergi Hydro-RP, 4 µm, 150 × 2.0 mm analytical column (Phenomenex, California, USA) with an isocratic mobile phase containing a mixture of acetonitrile, water, and formic acid (70:29.8:0.2) at a flow-rate of 300 µL/min. An AB Sciex API 3000 mass spectrometer at unit resolution in the multiple reaction monitoring mode was used to monitor the transition of the protonated precursor ions *m/z* 530.0 and *m/z* 539.1 to the product ions *m/z* 512.1 and *m/z* 521.3 for lumefantrine and the internal standard, respectively. Electrospray ionization was used for ion production [28, 29].

Day 7 lumefantrine concentrations greater than or equal to 0.2 μg/mL were considered therapeutic [30].

### Statistical analysis

Uncorrected efficacy was determined by dividing the number of treatment failures in each study arm by the total number of participants classified as either adequate clinical and parasitological response or treatment failure in that arm. Parasite clearance rates on day 2 and day 3 of follow-up were also examined to evaluate artemisinin delayed response.

Kaplan–Meier estimates of survival curves were calculated using the survival package in R version 3.3.2 (R Foundation for Statistical Computing, Vienna, Austria). Data from participants excluded for reasons other than treatment failure or incorrect enrollment were included in the analysis until the day of study departure.

To determine corrected therapeutic efficacy, microsatellite data were analysed using a previously published algorithm [31] that assigns each late treatment failure a posterior probability of recrudescence. A probability of more than 0.5 was considered a recrudescence, and less than or equal to 0.5 was designated as a reinfection.

The distribution of day 7 lumefantrine drug levels was compared between cases of adequate clinical and parasitological response and cases of recrudescence and reinfection using the Kolmogorov–Smirnov test. The proportion of participants with sub-therapeutic day 7 lumefantrine levels was compared between cases of adequate clinical and parasitological response and cases of recrudescence and reinfection using Fisher’s exact test.

## Results

### Triage and enrollment

Of 608 children enrolled in the study, 27 (4%) were lost to follow-up (loss to follow-up rates were 7% or less in all study arms), and 41 participants (7%) were excluded (Additional file 1). Participant characteristics at baseline reflected the different inclusion criteria in each site (Table 1). Median age ranged from 2.7 years in the Lunda Sul DP arm to 7.1 years in the Benguela DP arm. Sufficient participants completed follow-up to provide statistical power in all treatment arms, with between 85 and 94 participants reaching a primary study outcome in each arm.

**Table 1:** Number of participants enrolled and finishing follow-up and characteristics at baseline

	Benguela		Zaire		Lunda Sul	
	DP^b^	ASAQ^a^	AL^a^	ASAQ^a^	AL^a^	DP^b^
Enrollment and follow-up						
Enrolled, *n*	100	105	100	98	105	100
Lost to follow up, *n* (%)	5 (5)	2 (2)	3 (3)	5 (5)	5 (5)	7 (7)
Excluded, *n* (%)	10 (10)	12 (11)	3 (3)	3 (3)	9 (9)	4 (4)
Reached study endpoint, *n* (%)	85 (85)	91 (87)	94 (94)	90 (92)	91 (87)	89 (89)
Participant characteristics at baseline						
Median age, years (range)	7.1 (2–12)	6.3 (2–12)	3 (0.5–5)	2.7 (0.8–5)	3.3 (0.6–5)	3 (0.5–5)
Median weight, kg (range)	20 (9–48)	19 (10–52)	12 (7–20)	12 (7–19)	12 (6–20)	12 (6–19)
Percent female (%)	53	53	46	55	54	47
Median day 0 parasitaemia, parasites/µL (range)	25,940 (1218–95,585)	31,399 (1242–97,167)	47,607 (2175–182,138)	31,215 (2848–184,243)	22,340 (2162–122,064)	19,812 (3316–184,465)
Median day 0 haemoglobin, g/dL (range)	10.5 (8.1–13.9)	10.7 (8.1–15.1)	10.1 (8.1–13.0)	9.7 (8.1–13.0)	9.5 (8.1–12.8)	9.3 (8.1–14.9)

*AL* artemether–lumefantrine, *ASAQ* artesunate–amodiaquine, *DP* dihydroartemisinin–piperaquine

^a^28-day follow-up

^b^42-day follow-up

### Efficacy

Of 540 children completing follow up, 502 achieved adequate clinical and parasitological response, and 38 were classified as treatment failures (Table 2). Rates of parasite clearance were at least 97% on day 2 in all arms and 100% by day 3 in all arms (Table 3). No children experienced early treatment failure; all 38 treatment failures were late treatment failures.

**Table 2:** Treatment outcomes for participants finishing follow-up

	n (%)					
	Benguela		Zaire		Lunda Sul	
	DP^b^	ASAQ^a^	AL^a^	ASAQ^a^	AL^a^	DP^b^
	*n *= *85*	*n *= *91*	*n *= *94*	*n *= *90*	*n *= *91*	*n *= *89*
Treatment failure	5 (6)	1 (1)	7 (7)	16 (18)	9 (10)	0
Early treatment failure	0	0	0	0	0	0
Late treatment failure	5 (6)	1 (1)	7 (7)	16 (18)	9 (10)	0
Recrudescence	0	0	4 (4)	5 (6)	3 (3)	0
Day 21	0	0	1 (1)	5 (6)	3 (3)	0
Day 28	0	0	3 (3)	0	0	0
Day 35	0	–	–	–	–	0
Day 42	0	–	–	–	–	0
Reinfection	5 (6)	1 (1)	3 (3)	11 (12)	6 (7)	0
Day 21	0	0	0	7 (8)	2 (2)	0
Day 28	0	1 (1)	3 (3)	4 (4)	4 (4)	0
Day 35	2 (2)	–	–	–	–	0
Day 42	3 (4)	–	–	–	–	0
Adequate clinical and parasitological response	80 (94)	90 (99)	87 (93)	74 (82)	82 (90)	89 (100)

*AL* artemether–lumefantrine, *ASAQ* artesunate–amodiaquine, *DP* dihydroartemisinin–piperaquine

^a^28-day follow-up

^b^42-day follow-up

**Table 3:** Proportion of slides negative for asexual malaria parasites on days 2 and 3 following treatment

	Proportion slides negative (95% confidence intervals)					
	Benguela		Zaire		Lunda Sul	
	DP	ASAQ	AL	ASAQ	AL	DP
Day 2	98 (92–100)	100 (95–100)	97 (91–99)	97 (91–99)	97 (91–99)	100 (95–100)
Day 3	100 (95–100)	100 (95–100)	100 (95–100)	100 (95–100)	100 (95–100)	100 (95–100)

*AL* artemether–lumefantrine, *ASAQ* artesunate–amodiaquine, *DP* dihydroartemisinin–piperaquine

The uncorrected 28-day efficacy using the Kaplan–Meier estimate of the survival curve was 93% (88–98%) for AL in Zaire, 90% (84–96%) for AL in Lunda Sul, 99% (94–100%, 95% confidence interval) for ASAQ in Benguela, and 82% (75–90%) for ASAQ in Zaire (Table 4). The uncorrected 42-day efficacy was 95% (90–99%) for DP in Benguela and 100% (100–100%) for DP in Lunda Sul.

**Table 4:** Efficacy of first-line anti-malarials in three therapeutic efficacy monitoring sites in Angola, 2017

	Efficacy (95% confidence intervals)					
	Benguela		Zaire		Lunda Sul	
	DP^b^	ASAQ^a^	AL^a^	ASAQ^a^	AL^a^	DP^b^
Uncorrected						
Per-protocol day 28	100 (95–100)	99 (94–100)	92.8 (85–97)	82.2 (72–89)	90.1 (82–95)	100 (95–100)
Per-protocol day 42	94.6 (87–98)	–	–	–	–	100 (95–100)
Kaplan–Meier estimate day 28	100 (100–100)	98.9 (97–100)	92.7 (88–98)	82.1 (75–90)	90.1 (84–96)	100 (100–100)
Kaplan–Meier estimate day 42	94.6 (90–99)	–	–	–	–	100 (100–100)
PCR-corrected						
Per-protocol day 28	100 (97–100)	100 (97–100)	95.5 (89–98)	93 (85–97)	96.4 (90–99)	100 (96–100)
Per-protocol day 42	100 (96–100)	–	–	–	–	100 (96–100)
Kaplan–Meier estimate day 28	100	100 (97–100)	95.5 (91–100)	93.3 (88–99)	96.5 (93–100)	100
Kaplan–Meier estimate day 42	100 (96–100)	–	–	–	–	100

Per-protocol efficacy defined as proportion adequate clinical and parasitological response, Kaplan–Meier estimate calculated from estimate of survival function

*AL* artemether–lumefantrine, *ASAQ* artesunate–amodiaquine, *DP* dihydroartemisinin–piperaquine

^a^28-day follow up

^b^42-day follow up

The majority of the 38 late treatment failures were reinfections (26, 68%) rather than recrudescences (12, 32%) by microsatellite analysis (Table 2). Late treatment failures were categorized with a high degree of confidence, with the posterior probability of recrudescence greater than 90% or less than 10% in all but three instances (Additional file 2). One late treatment failure in the Zaire AL arm had a posterior probability of recrudescence of 29%, and two late treatment failures in the Zaire ASAQ arms had probabilities of 19 and 39%. Four cases of recrudescence were identified in the Zaire AL arm, three in the Lunda Sul AL arm, and five in the Zaire ASAQ arm. No recrudescences were found among participants in the Benguela DP, Lunda Sul DP, or Benguela ASAQ arms.

After exclusion of reinfections, the corrected 28-day efficacy of AL was 96% (91–100%), in Zaire and 97% (93–100%) in Lunda Sul. The corrected 28-day efficacy of ASAQ was 100% (97–100%) in Benguela and 93% (88–99%) in Zaire. The corrected 42-day efficacy of DP was 100% (96–100%) in Benguela and 100% in Lunda Sul.

### Safety

Among the 586 participants enrolled within inclusion criteria, 11 participants (2%) reported adverse medication effects: four with vomiting after DP (2% of participants in DP arms), three with nausea after ASAQ (2%), two with excessive sweating after ASAQ (1%), one with vomiting after ASAQ (1%), and one with excessive sweating after DP (1%). No participants reported adverse effects after AL administration, neither under direct observation nor at home. No participants left the study due to adverse medication effects.

### Molecular markers of resistance

Almost all (75/76, 99%) of the day 0 and day of failure samples analysed from the 38 treatment failures were wild-type for *pfk13* (Table 5). One sample from Zaire exhibited the A504V mutation, which has not been associated with artemisinin resistance. Nearly all day of failure samples in the AL arms carried the *pfcrt* wildtype K76 genotype (14/16, 88%), and all had the N86 *pfmdr1* genotype (16/16, 100%). All ASAQ day of failure samples (17/17, 100%) carried the *pfcrt* 76T single-nucleotide polymorphism. The only two samples with the *pfmdr1* 86Y mutation were ASAQ day of failure samples from Benguela.

**Table 5:** Molecular markers of resistance for treatment failures observed during therapeutic efficacy monitoring in Angola, 2017

Marker	n (%)															
	Benguela				Zaire								Lunda Sul			
	DP		ASAQ		AL				ASAQ				AL			
	Reinf day 0	Reinf day failure	Reinf day 0	Reinf day failure	Reinf day 0	Reinf day failure	Recr day 0	Recr day failure	Reinf day 0	Reinf day failure	Recr day 0	Recr day failure	Reinf day 0	Reinf day failure	Recr day 0	Recr day failure
	*n *= *5*	*n *= *5*	*n *= *1*	*n *= *1*	*n *= *3*	*n *= *3*	*n *= *4*	*n *= *4*	*n *= *11*	*n *= *11*	*n *= *5*	*n *= *5*	*n *= *6*	*n *= *6*	*n *= *3*	*n *= *3*
*pfk13* sequence																
Wildtype	5 (100)	5 (100)	1 (100)	1 (100)	2 (67)	3 (100)	4 (100)	4 (100)	11 (100)	11 (100)	5 (100)	5 (100)	6 (100)	6 (100)	3 (100)	3 (100)
A504V	–	–	–	–	1 (33)	–	–	–	–	–	–	–	–	–	–	–
*pfcrt* sequence^a^																
CVMNK	2 (40)	4 (80)	–	–	1 (33)	3 (100)	1 (25)	3 (75)	5 (45)	–	2 (40)	–	6 (100)	5 (83)	3 (100)	3 (100)
CVIET	4 (80)	1 (20)	1 (100)	1 (100)	2 (67)	–	3 (75)	2 (50)	7 (64)	11 (100)	5 (100)	5 (100)	1 (17)	1 (17)	–	–
*pfmdr1* sequence^b^																
NYD	4 (80)	5 (100)	1 (100)	1 (100)	3 (100)	2 (67)	4 (100)	3 (75)	5 (45)	7 (64)	4 (80)	5 (100)	6 (100)	5 (83)	2 (67)	2 (67)
NFD	1 (20)	–	–	–	–	1 (33)	–	1 (25)	6 (55)	2 (18)	1 (20)	–	–	1 (17)	2 (67)	2 (67)
YFD	–	–	–	–	–	–	–	–	–	2 (18)	–	–	–	–	–	–
*pfmdr1* copy number																
1	5 (100)	5 (100)	1 (100)	1 (100)	3 (100)	3 (100)	4 (100)	4 (100)	11 (100)	11 (100)	5 (100)	5 (100)	6 (100)	6 (100)	3 (100)	3 (100)
*plasmepsin 2* copy number																
1	5 (100)	5 (100)	1 (100)	1 (100)	3 (100)	3 (100)	4 (100)	4 (100)	11 (100)	11 (100)	5 (100)	5 (100)	6 (100)	6 (100)	3 (100)	3 (100)

*Reinf* reinfection, *Recr* recrudescence, *AL* artemether–lumefantrine, *ASAQ* artesunate–amodiaquine, *DP* dihydroartemisinin–piperaquine

^a^*pfcrt* haplotype classified according to amino acids at positions 72, 73, 74, 75, and 76; mixed infections were included in the numerator for each haplotype

^b^*pfmdr1* haplotype classified according to amino acids at positions 86, 184, and 1246; mixed infections were included in the numerator for each haplotype

All 76 analysed samples had only one copy of both the *pfmdr1* and *pfpm2* genes. However, 75 of 76 samples contained multiple *P. falciparum* strains.

### Lumefantrine concentrations

Median day 7 lumefantrine concentrations in the Zaire AL arm were similar among participants experiencing adequate clinical and parasitological response, reinfection, and recrudescence, ranging from 0.19 to 0.23 μg/mL (Additional files 3, 4). Two (50%) out of the four cases of recrudescence observed in the AL arm had sub-therapeutic day 7 lumefantrine levels, but this did not differ significantly (p value 1.0) from rate of sub-therapeutic day 7 lumefantrine levels in participants with adequate clinical and parasitological response (35/87, 40%). Two children had undetectable lumefantrine levels, one of whom successfully responded to treatment and one of whom had a recrudescent infection. The distribution of day 7 lumefantrine concentrations was not statistically different between cases of adequate clinical and parasitological response and recrudescence (p value 0.94), between cases of adequate clinical and parasitological response and reinfection (p value 0.34), or between cases of adequate clinical and parasitological response and recrudescence/reinfection (0.80).

## Discussion

AL, ASAQ, and DP remain efficacious for the treatment of uncomplicated *P. falciparum* malaria in the sites where they were tested. The WHO recommends changing national malaria treatment policy if efficacy drops below 90% [2], and all corrected efficacies reported here exceed that threshold. All three medications were very well tolerated, with less than 2% of participants experiencing mild adverse effects and no participants experiencing serious adverse effects.

Uncorrected therapeutic efficacy was above 90% in all study arms except ASAQ in Zaire, which had an uncorrected efficacy of 82%. Low uncorrected efficacy has been noted in previous anti-malarial studies in Zaire [6, 7] and may be explained in part by relatively high malaria transmission in this province as compared to the other sites.

There has been concern recently around possible lumefantrine resistance in Angola, particularly in Zaire. The results presented here do not support this concern, with corrected AL efficacy at 96% in Zaire and 97% in Lunda Sul. Greater efforts to administer all AL doses with a fatty meal could explain the higher lumefantrine efficacy observed in this round of monitoring. Nevertheless, a substantial proportion (40%) of cases of adequate clinical and parasitological response had day 7 lumefantrine concentrations below the recommended therapeutic threshold of 0.2 μg/mL. There was no statistically significant difference in day 7 lumefantrine drug levels by treatment outcome in the AL Zaire arm, but this could be due to the small number of treatment failures observed in that arm.

DP demonstrated the highest efficacy of all anti-malarials in this study, with no recrudescences identified throughout 42 days of follow-up among 174 participants taking this medication. This high efficacy, combined with the drug’s relatively long half-life, confirms DP as an appropriate ACT for Angola [32]. Although the utility of this medication is declining in Asia, DP efficacy remains high throughout Africa [33].

Multiple factors provide reassurance that *P. falciparum* is still highly susceptible to artemisinin derivatives in Angola. No early treatment failures were observed in this study. Parasite clearance was rapid in all study arms, with parasitaemia resolving in at least 97% of participants by day 2 of follow-up and all participants by day 3. Finally, no mutations in *pfk13* associated with artemisinin resistance were identified during molecular analysis of treatment failures.

In contrast to the previous rounds of therapeutic efficacy studies [6, 7], the minimum haemoglobin required for inclusion was increased to 8 g/dL from 5 g/dL, possibly explaining why no early treatment failures were observed in the current round. All but one of the early treatment failures observed in both the 2013 and 2015 studies were due to severe anemia. A haemoglobin of less than 5 g/dL in the presence of parasitaemia is a sign of severe malaria and, if observed during follow up, would prompt concern for treatment failure. However, it is common for patients on anti-malarial treatment to experience an initial drop in haemoglobin before improving. Children enrolled with a haemoglobin near 5 g/dL who undergo this customary decrease may be inappropriately classified as early treatment failures, when in fact they were following the expected treatment course.

Analysis of markers of partner drug resistance in AL and ASAQ treatment failures yielded results consistent with previous understanding of these resistance markers. Mutations in *pfcrt* K76 and *pfmdr1* N86 are known to be associated with AL resistance [17], and these were present on day of failure in nearly all AL treatment failures. The *pfcrt* K76T genotype, which has been associated with decreased ASAQ susceptibility [17], was identified in all ASAQ treatment failures from this study. The only instances of a *pfmdr1* 86Y mutation, associated with amodiaquine resistance, were identified in ASAQ treatment failures.

As AL and ASAQ exhibit opposite selective pressures on certain loci in the *pfmdr1* and *pfcrt* genes, alternating the use of these drugs might preserve diversity in these genes and minimize the emergence of resistance. Analysis of day 0 samples from the 2015 therapeutic efficacy study has shown that the majority of parasites circulating in the three sentinel provinces in Angola carry the *pfmdr1* N86 genotype [34]. A similar analysis of all day 0 samples from this current study could be useful in characterizing how the circulating parasite population is affected by the selective pressures on the *pfmdr1* gene.

None of the day 0 or day of failure samples had an elevated copy number of *pfmdr1*, consistent with the general absence [35, 36] or low rates [37, 38] of *pfmdr1* copy number variation in sub-Saharan Africa. As a recently proposed marker, there are few available data on *pfpm2* copy number variation in sub-Saharan Africa; absence of elevated copy number in this gene in the samples analysed here is consistent with relatively low rates of DP use in sub-Saharan Africa. However, all but one of the samples analysed for copy number variation contained multiple *P. falciparum* strains, so this analysis of could have missed an elevated copy number in parasites occurring at low-frequencies in mixed-strain infections.

Although the study was carried out in three provinces of Angola with varied geography and malaria endemicity, it is possible that these results may not be representative of either any one province or the entire country. Furthermore, since assignment of treatment drug was not random, drug efficacies should not be compared across study arms.

## Conclusions

All three evaluated artemisinin-based combinations continue to be efficacious against *P. falciparum* malaria in the three sentinel sites in Angola. Rapid parasite clearance is consistent with full susceptibility to artemisinin derivatives. Periodic monitoring of in vivo drug efficacy remains a critical routine activity for the Angolan NMCP.

## Additional files

**Additional file 1.** Consort flow diagrams for follow-up outcomes in all six study arms, 2017.

**Additional file 2.** Microsatellite marker results, Angola therapeutic efficacy monitoring, 2017.

**Additional file 3.** Day 7 lumefantrine drug levels in patients treated with artemether-lumefantrine, by treatment outcome, Zaire, Angola.

**Additional file 4.** Day 7 lumefantrine levels in patients treated with artemether-lumefantrine, by treatment outcome, Zaire, Angola.
