# Supplementary material for: Efficacy and safety of artemether–lumefantrine, artesunate–amodiaquine, and dihydroartemisinin–piperaquine for the treatment of uncomplicated Plasmodium falciparum malaria in three provinces in Angola, 2017
Source: Malar J. 2018 Apr 3;17:144. doi: 10.1186/s12936-018-2290-9 (PMC5883595; doi:10.1186/s12936-018-2290-9)
Supplement: Supplementary file 4 — Additional file 4. Day 7 lumefantrine levels in patients treated with artemether-lumefantrine, by treatment outcome, Zaire, Angola. [file 12936_2018_2290_MOESM4_ESM.docx]

**Additional file 4**. Day 7 lumefantrine levels in patients treated with artemether-lumefantrine, by treatment outcome, Zaire, Angola.

The concentration for two samples below limit of quantification was treated as half the limit of quantification. There was no statistically significant difference between the distribution of Day 7 lumefantrine drug levels between cases of ACPR and recrudescence (Kolmogorov–Smirnov test p-value 0.94), between cases of ACPR and reinfection (p-value 0.34), or between cases of ACPR and recrudescence/reinfection (0.80). ACPR: Adequate clinical and parasitological response; Recr: Recrudescence; Reinf: Reinfection.
