# Supplementary material for: Efficacy and safety of artemether–lumefantrine, artesunate–amodiaquine, and dihydroartemisinin–piperaquine for the treatment of uncomplicated Plasmodium falciparum malaria in three provinces in Angola, 2017
Source: Malar J. 2018 Apr 3;17:144. doi: 10.1186/s12936-018-2290-9 (PMC5883595; doi:10.1186/s12936-018-2290-9)
Supplement: Supplementary file 4 [file 12936_2018_2290_MOESM1_ESM.docx]

BENGUELA

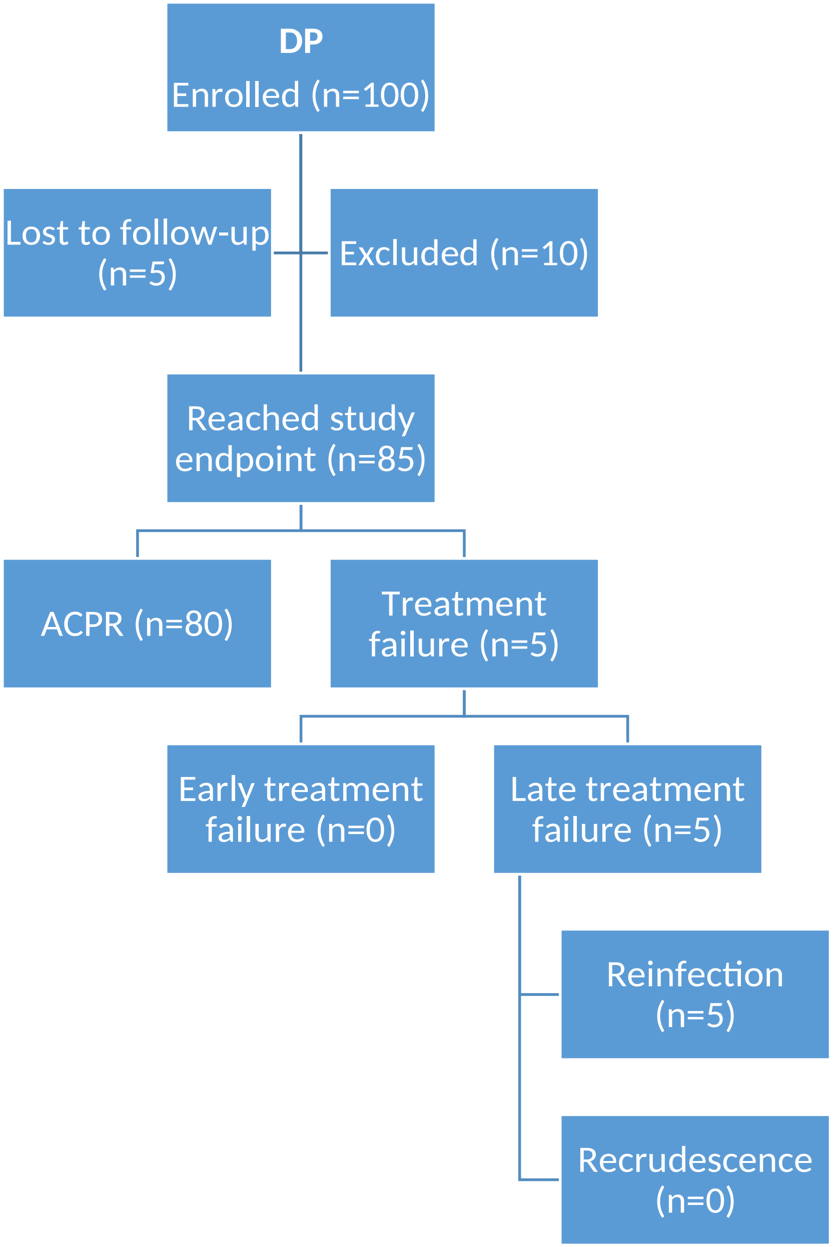
DP
Enrolled (n=100)
Lost to follow-up (n=5)
Excluded (n=10)
Reached study endpoint (n=85)
ACPR (n=80)
Treatment failure (n=5)
Early treatment failure (n=0)
Late treatment failure (n=5)
Reinfection (n=5)
Recrudescence (n=0)

ZAIRE

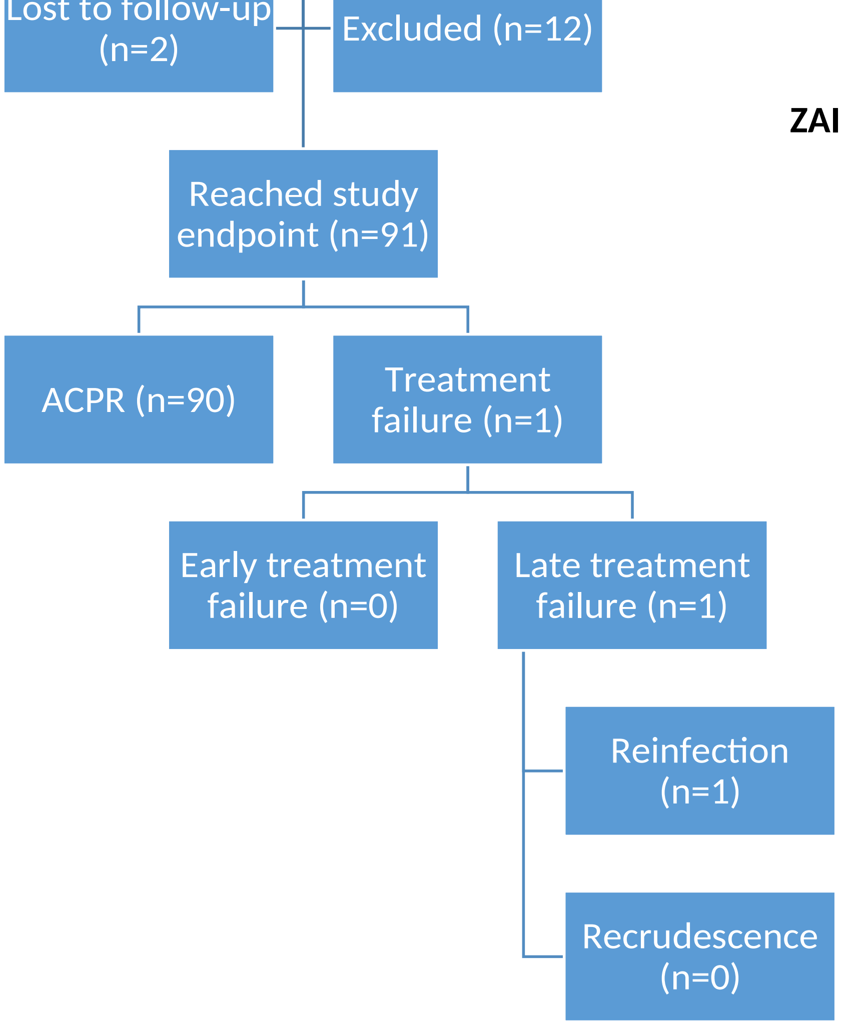

Lost to follow-up (n=2)
Excluded (n=12)
Reached study endpoint (n=91)
ACPR (n=90)
Treatment failure (n=1)
Early treatment failure (n=0)
Late treatment failure (n=1)
Reinfection (n=1)
Recrudescence (n=0)

LUNDA SUL

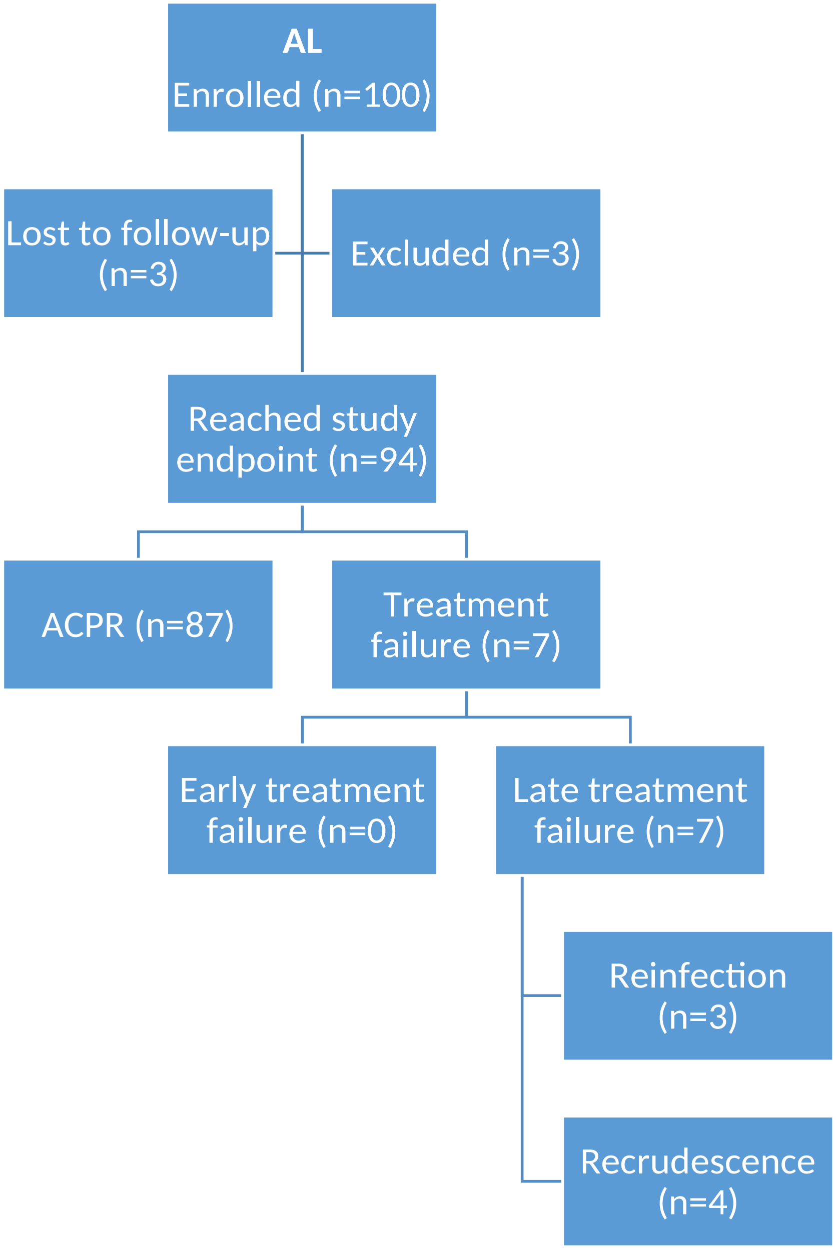

AL: artemether-lumefantrine; ASAQ: artesunate-amodiaquine; DP: dihydroartemisinin-piperaquine; ACPR: adequate clinical and parasitological response

ZAIRE

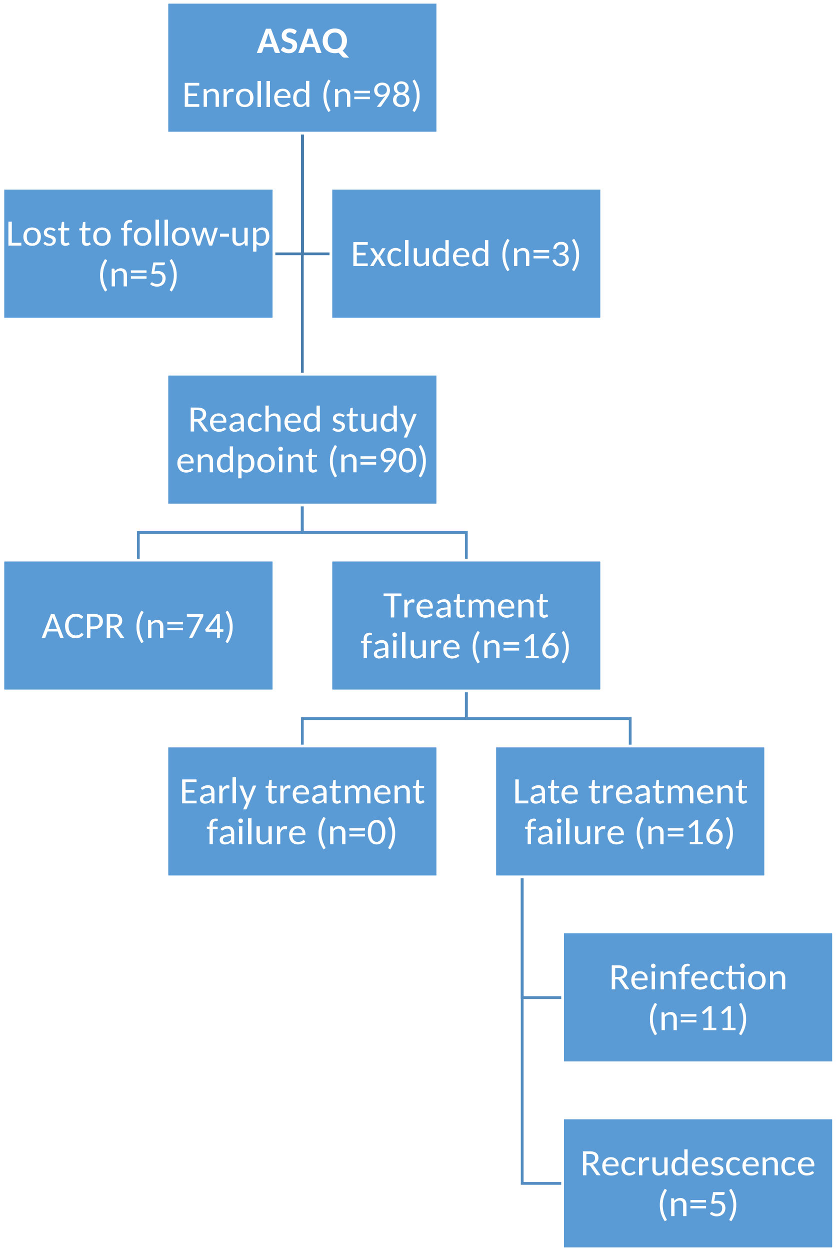
ASAQ
Enrolled (n=98)
Lost to follow-up (n=5)
Excluded (n=3)
Reached study endpoint (n=90)
ACPR (n=74)
Treatment failure (n=16)
Early treatment failure (n=0)
Late treatment failure (n=16)
Reinfection (n=11)
Recrudescence (n=5)

LUNDA SUL

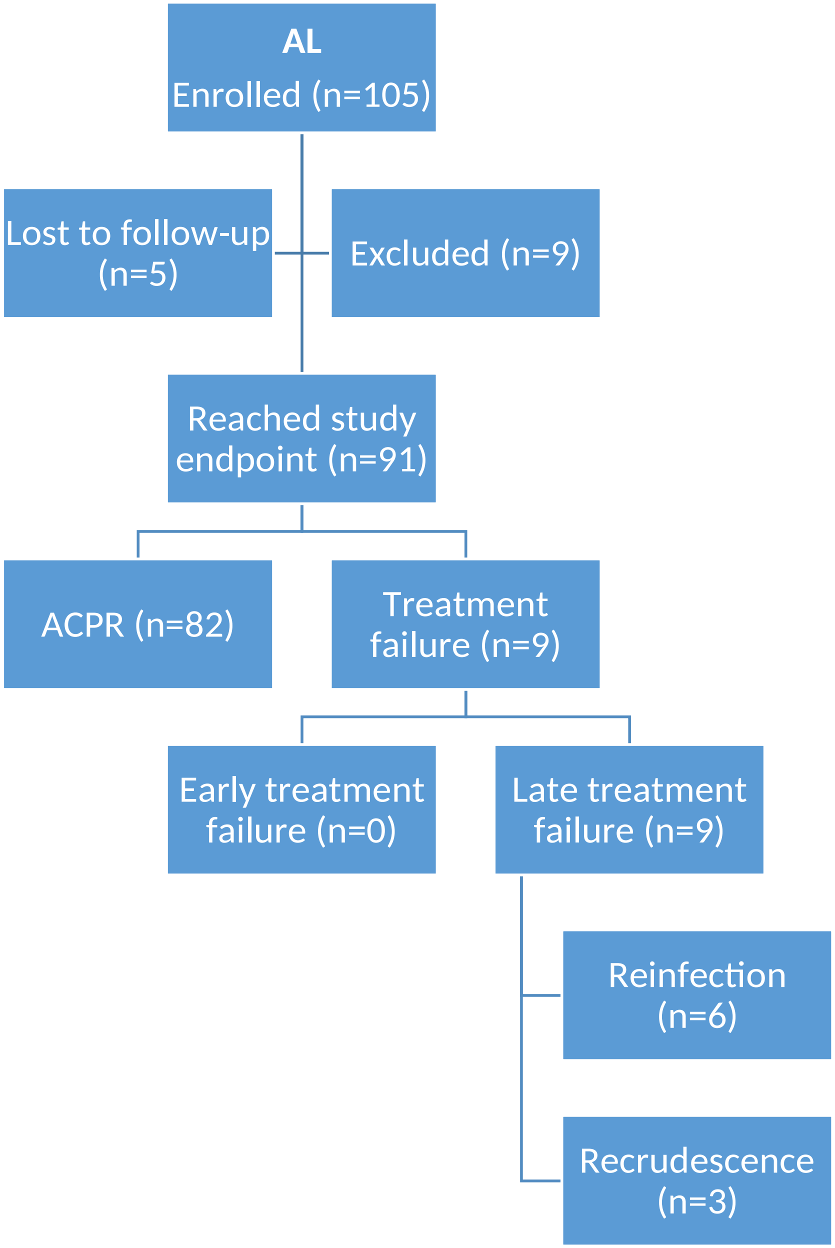

AL: artemether-lumefantrine; ASAQ: artesunate-amodiaquine; DP: dihydroartemisinin-piperaquine; ACPR: adequate clinical and parasitological response

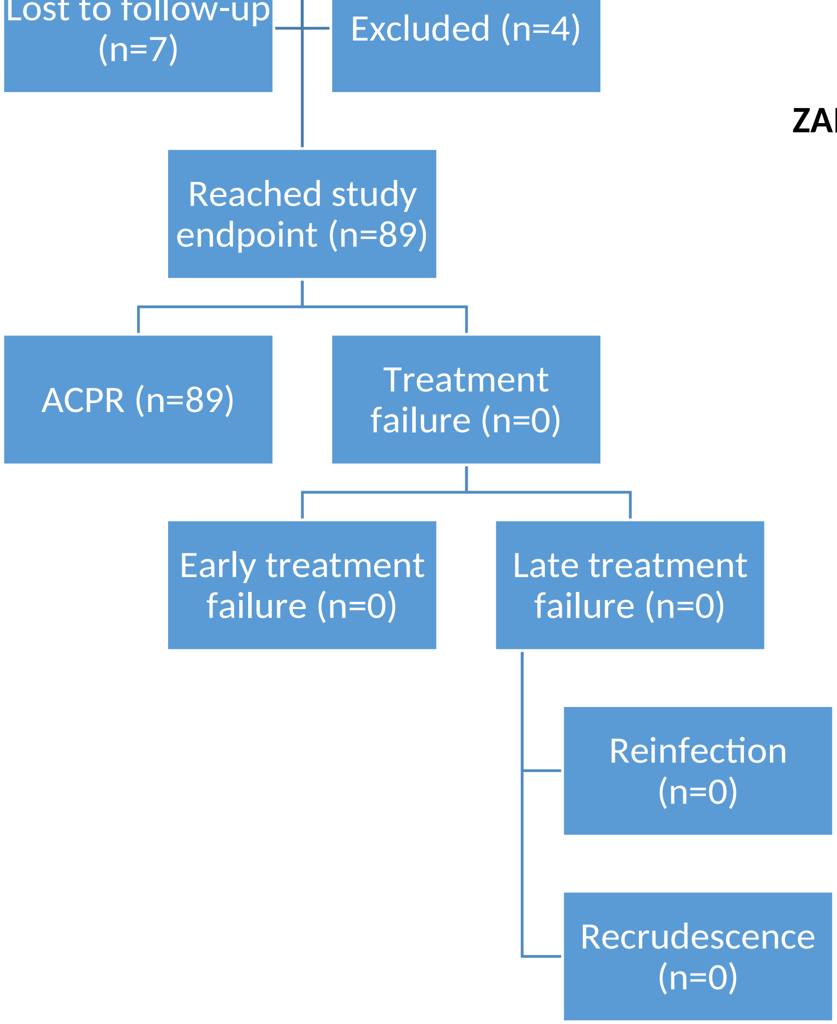
Lost to follow-up (n=7)
Excluded (n=4)
Reached study endpoint (n=89)
ACPR (n=89)
Treatment failure (n=0)
Early treatment failure (n=0)
Late treatment failure (n=0)
Reinfection (n=0)
Recrudescence (n=0)

ZAIRE
